# Preventive measures for Parkinson’s disease: insights into motivation and barriers from the patients’ perspective

**DOI:** 10.1186/s12883-026-04703-0

**Published:** 2026-02-11

**Authors:** Julienne Haas, Annette Rogge, Birte Otto, Nathalie Michel, Daniela Berg, Eva Schaeffer

**Affiliations:** 1 Department of Neurology, Christian-Albrechts-University, Arnold-Heller-Str. 3, Kiel, 24105 Germany; 2 Department of Neurology, Nordseeklinik Helgoland, Helgoland, Germany

**Keywords:** Parkinson’s disease, Diet, Exercise, Preventive measures

## Abstract

**Background:**

The prevalence of Parkinson’s disease (PD) is increasing globally and to date there is no disease-modifying pharmacological treatment. In recent years, however, a solid evidence base has been established that lifestyle changes, including physical activity and Mediterranean diet, can have a disease-modifying effect. Nevertheless, there are currently no standardized lifestyle intervention programs and the opinion of PD patients on lifestyle changes as a possible form of therapy is not clear. Therefore, the aim of this study was to investigate whether PD patients consider lifestyle changes to be relevant for their disease, and which hindering and supporting factors they describe when implementing preventive measures.

**Methods:**

Patients were recruited from two German neurology clinics. A standardized questionnaire was used to evaluate the patients’ perception of education and implementation of lifestyle changes.

**Results:**

The study included 107 PD patients. Most patients stated that they tried to implement recommendations on lifestyle changes (*n* = 89, 85%) and almost all considered education about lifestyle changes to be important (*n* = 96, 94%). Supporting factors for implementation included prevention programs, bonus programs as well as digital approaches. Obstacles included the fact that patients were only able to maintain their motivation for a short time, lack of knowledge and lack of cooperation from partners as well as physical limitations.

**Conclusion:**

PD patients seem to be very interested in lifestyle changes and try to implement them but encounter various obstacles. Structured support programs could help maintain lifestyle changes and pave the way for effective non-pharmacological disease modification.

**Supplementary Information:**

The online version contains supplementary material available at 10.1186/s12883-026-04703-0.

## Background

The prevalence of Parkinson’s disease (PD) is increasing globally and is associated with a high individual burden as well as rising socio-economic costs [1, 2]. As a consequence, there is an urgent need for disease-modifying therapeutic interventions. So far, however, no disease-modifying pharmacological treatment has found its way into clinical practice. In recent years there has therefore been an increasing focus on non-pharmacological preventive measures, particularly on lifestyle changes, which can be implemented in healthy individuals but also in the prodromal phase of PD to positively influence the course of the disease already at this stage. In this context, there is already substantial evidence that, among others, diet and physical activity can reduce the risk of PD on the one hand and have a disease-modifying effect in the clinical course of the disease on the other hand. The most convincing evidence to date has been provided for physical activity (especially moderate endurance training) and the Mediterranean diet. These lifestyle adjustments have been associated with a risk reduction of up to 60% regarding the development of PD (primary prevention) [3–6]. Moreover, several epidemiological studies indicate that lifestyle factors may influence the timing of disease onset. Greater adherence to a Mediterranean or MIND dietary pattern, as well as regular engagement in moderate levels of physical activity, has been linked to a later age at onset of the disease (indicating a potential secondary preventive effect) [7–10]. Finally, several studies using diverse exercise programs with follow-up periods of at least 24 months have shown clinically meaningful and lasting improvements in motor function, suggesting potential disease-modifying effects, while one large retrospective analysis found that higher levels of self-reported moderate to vigorous physical activity were associated with fewer motor impairments after a median follow-up of five years (tertiary prevention) [11–13].

Based on these observations, individual clinics and doctors have already begun to integrate advice on lifestyle factors into everyday clinical practice on their own initiative. In addition, patient initiatives and support groups have begun to promote lifestyle changes. However, there are currently no standardized lifestyle intervention programs and no recommendations on how patients can be educated and supported regarding the implementation of lifestyle changes. Additionally, it is not known whether and to what extend patients would accept lifestyle changes as a form of therapy (therapy acceptance) and adhere to these lifestyle changes over a longer period of time (therapy adherence). Patients’ attitudes towards a treatment or therapy depends on the benefit they experience or expect from it, as opposed to potential barriers or disadvantages they are concerned to overcome [14]. However, it is currently unclear which barriers PD patients consider and possibly even fear. Exploring them is of great importance, since sustainable disease-modifying effects can only be expected through long-term lifestyle changes.

The aim of this study was to investigate (i) whether PD patients consider lifestyle changes and information about these as relevant, (ii) the factors patients consider as relevant to maintain lifestyle measures in the long term and (iii) what barriers they see that may prevent them from changing their lifestyle.

## Methods

### Questionnaire

In close interdisciplinary collaboration (neurology and clinical ethics), a standardized questionnaire to evaluate patients’ perception on education and implementation of lifestyle changes was designed. Questions and possible answers were based on clinical experience and literature research on the topic. The questionnaire included information about sex, age, diagnosis of PD and disease onset as well as comorbidities and medication. Regarding education and implementation of lifestyle changes, the questionnaire comprised the following questions:

- whether patients were informed by a doctor about the benefit of lifestyle changes (single selection, yes or no)
- who exactly informed them (multiple selection, e.g. family doctor or specialist)
- what they were informed about (multiple selection, e.g. exercise or nutrition)
- whether they have changed their behavior (multiple selection; e.g. yes, no or only in parts)
- how important they would rate education on lifestyle changes (Likert-Scale: -3, not important to 3, very important)
- how and by whom PD patients should be informed about lifestyle changes (multiple selection, e.g. public talks, at the neurologist’s appointment)
- what would help them to implement lifestyle changes (multiple selection, e.g. including partner, digital solutions, advise from neurologist)
- as well as which factors prevented them from changing their lifestyle (multiple selection, e.g. lack of knowledge, lack of motivation)

Six of these questions allowed additional free text answers.

A detailed description of the questionnaire can be found in the additional files (Additional file 1, translated version).

### Recruitment

Recruitment took place between August 2023 and December 2024. PD patients were recruited from the Department of Neurology in Kiel (specialized outpatient clinic for the early detection of PD) and the Nordseeklinik Helgoland (specialized clinic for the care of individuals with PD, including patients with a longer disease duration). The routine clinical care of patients at these two facilities includes education about possible disease-modifying effects of lifestyle changes.

Inclusion criteria were: (1) diagnosis of PD according to the Movement Disorder Society clinical diagnostic criteria for PD [15] and (2) ability to give informed consent. Exclusion criteria included: (1) diagnosis of other clinically significant neurological diseases.

### Statistic

Characteristics were tested for normal distribution using the Shapiro-Wilk test. Mean value and standard deviation were calculated for normally distributed variables. For not normally distributed variables median and range were calculated. Calculations were done using R (Version 2024.09.1 + 394) and SPSS Statistics 30 (IBM). To examine potential associations between clinical parameters (gender, age, duration of illness) and the participants’ responses to the items on potential barriers and supportive factors, binary logistic regression analyses were performed for each dichotomous outcome variable. A significance level of *p* < 0.05 was applied.

### Ethics

This study was approved by the local ethics committee of the University of Kiel and all participants gave written informed consent (ethic vote number D560/23 and 058/23m).

## Results

### Clinical characteristics

107 PD patients took part in the study. One questionnaire could not be evaluated due to incompleteness. All participants gave their consent to data storage and processing as well as anonymized publication of the results. Mean age was 65 years; 41 (38%) of the participants were female. Median disease duration since diagnosis was 4 years; median levodopa equivalent dose was 465 mg. 65 patients (61%) reported taking a dopamine agonist while 89 (83%) reported levodopa intake. Demographic and clinical data are shown in Table 1.

**Table 1:** Characteristics

	PD
*N*	107
Female gender (n/%)	41 (38%)
Age (years)	65 (9)^a^
Disease duration since diagnosis (years)	4 (0–26)^b^
Levodopa intake (yes/%)	89 (83%)
Dopamine agonist intake (yes/%)	65 (61%)
Antidepressants intake (yes/%)	12 (11%)
Levodopa Equivalent Dose (in mg)	465 (0-1815)^b^

^a^Mean (Standard deviation)

^b^Median (Range)

### Education about lifestyle changes in PD

The majority of patients with PD stated that they had been informed by a doctor about the positive effects of lifestyle changes (*n* = 94, 88%). Some stated that they received information from other professional groups or support groups only or in addition to the information they received from their doctor (*n* = 15, 14%). Most patients were informed by their neurologist (*n* = 74, 69%) or in the above-mentioned specialized PD out- as well as inpatient clinic (*n* = 36, 34% and *n* = 46, 43%). A few were informed by their family doctor (*n* = 26, 24%). Most patients were informed about the positive effects of physical activity on the course of the disease (*n* = 93, 87%). Many were also informed about the effects of nutrition (*n* = 70, 65%). In addition to lifestyle changes, the majority of patients were also informed about the possible beneficial effects of guided physical therapy forms as a symptom-oriented therapy of PD (*n* = 82, 77%).

### Implementation of lifestyle changes in PD

**Fig. 1:**
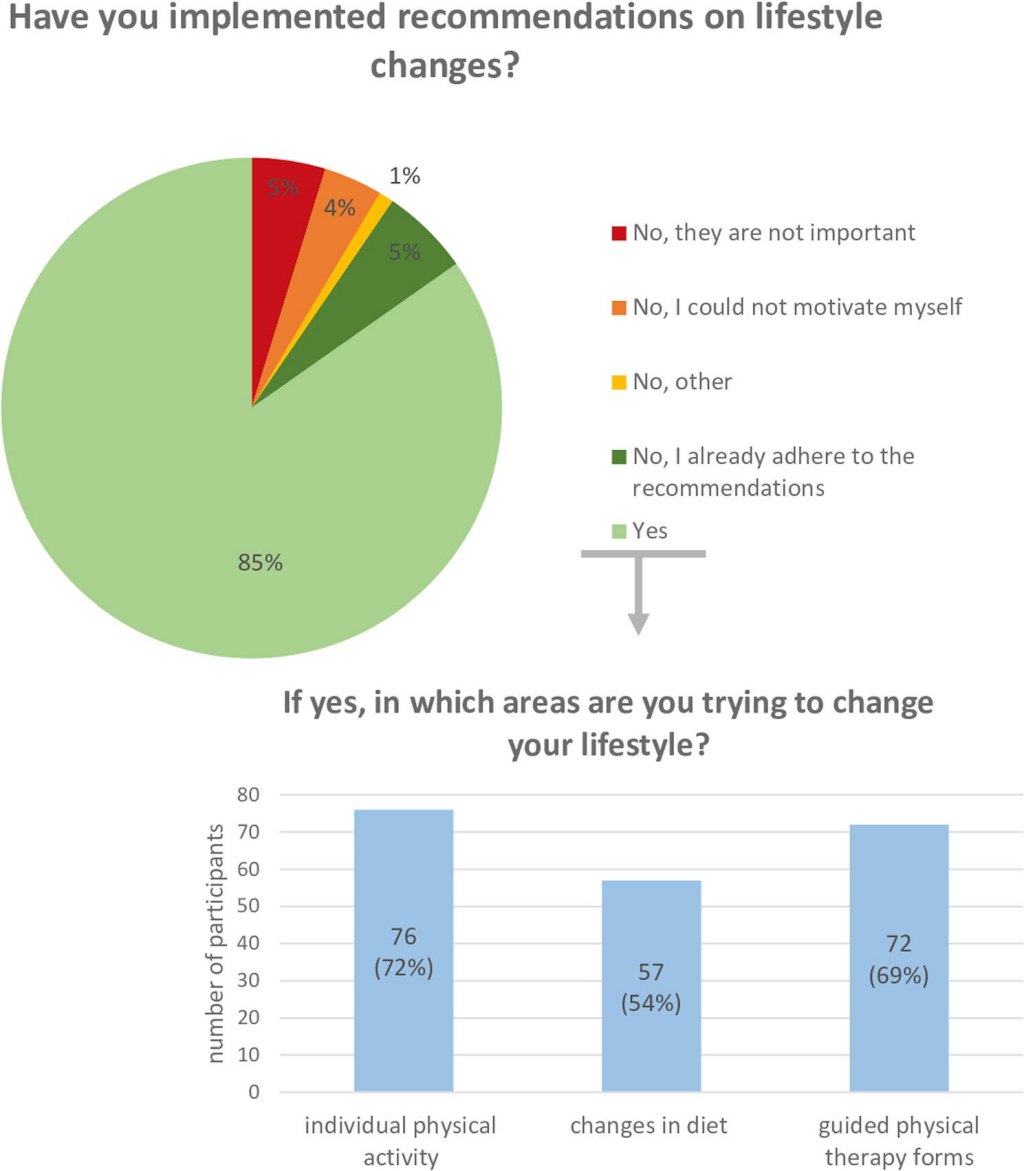
Implementation of recommendations, multiple answers possible regarding areas of lifestyle changes

Most patients stated that they tried to implement recommendations on lifestyle changes (*n* = 89, 85%). A minority of patients had already followed this lifestyle before diagnosis and treatment (*n* = 6, 6%). Only a small group stated that they did not want to implement recommendations on lifestyle changes due to a lack of interest or motivation (*n* = 10, 10%) (see Fig. 1).

After receiving information on lifestyle changes, many patients stated that they were paying more attention to individual physical activity (*n* = 76, 72%) as well as nutrition (*n* = 57, 54%). More than two thirds of the PD patients tried to integrate guided physical therapy forms into their daily lives (*n* = 72, 69%) (see Fig. 1).

### Importance of education

**Fig. 2:**
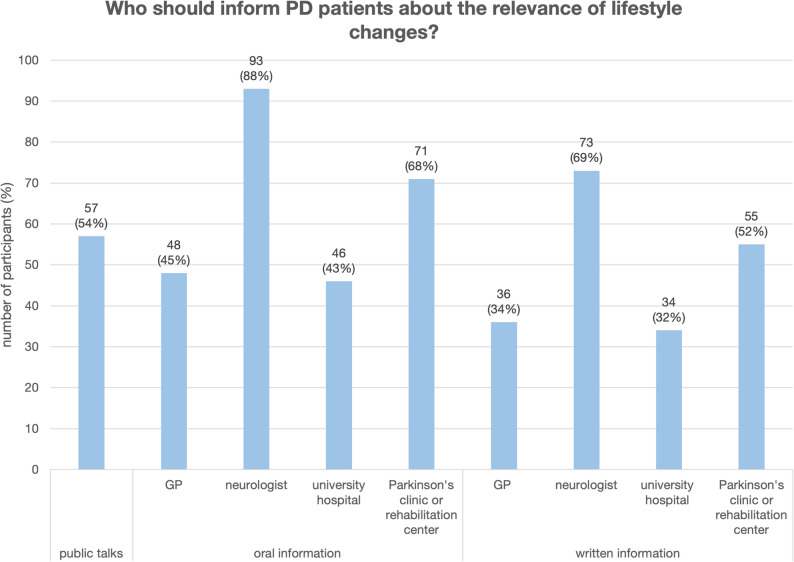
Information about lifestyle changes; Notes: GP, general practitioner

Almost all PD patients considered education about the possible positive aspects of lifestyle changes on the course of the disease to be important or very important (*n* = 96, 94%). A total of 84 patients stated that they considered information about lifestyle changes to be very important (82%, Likert-scale + 3). Twelve patients considered it to be important (12%, Likert-scale + 2). One patient considered it slightly important (1%, Likert-scale + 1), four patients were neutral (4%, Likert-scale 0), and one stated that they considered the information slightly unimportant (1%, Likert-scale − 1). No patients considered it unimportant or very unimportant (0%, Likert-scale − 2 and − 3). PD patients stated that they would like to be informed by their neurologist (oral information: *n* = 93, 88%; written information: *n* = 73, 69%) or as part of a presentation at a PD specialist or rehabilitation clinic (oral information: *n* = 71, 67%; written information: *n* = 55, 52%). Fewer PD patients stated that they would like to be informed by their family doctor (oral information: *n* = 48, 45%; written information: *n* = 36, 34%) or at public events (*n* = 57, 54%) (see Fig. 2). The question was designed as a multiple response question, with the majority of respondents stating that they would like information from several representatives of the healthcare system. For public talks and oral information, 87% of the patients surveyed gave at least 2 answers; for written information, 63% wanted information from at least 2 different sources.

### Supporting factors for implementation of lifestyle changes

**Fig. 3:**
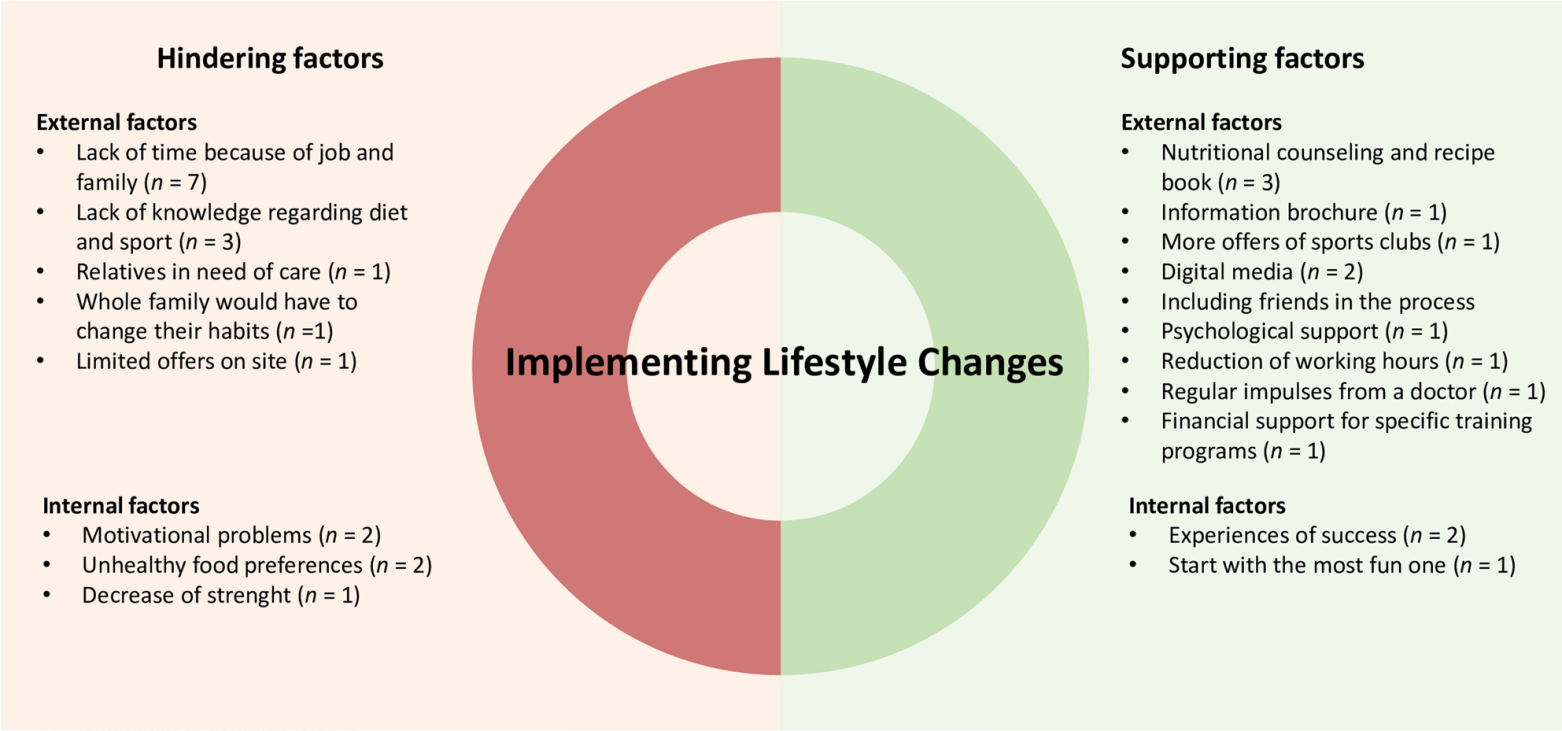
Hindering and supporting factors in the implementation of lifestyle changes in *n* = 107 individuals with PD. Free text answers

Patients thought of various factors that could help them to implement lifestyle changes. This included implementation of prevention programs (*n* = 58, 54%), e.g. a supervised sports program, bonus programs (health insurance programs that reward health-promoting behavior, such as the promotion of measures to achieve a standardized body mass index, membership in a sports club and participation in preventive medical check-ups) (*n* = 40, 37%) as well as digital approaches (*n* = 50, 47%). Additionally, many PD patients indicated that they would like individual advice from their neurologist (*n* = 80, 75%). Some also stated that they would like advice from their family doctor (*n* = 32, 30%). Also, many PD patients reported that they would like their partner to be included in information about implementing lifestyle changes (*n* = 65, 61%). Free-text responses (see Fig. 3) contained various supporting factors, which were divided into external and internal factors. Patients mentioned helpful external factors such as nutritional counseling (*n* = 3, 3%), more opportunities for physical activity (*n* = 1, 1%), the involvement of the media, doctors, and friends (*n* = 4, 4%), as well as a reduction in working hours (*n* = 1, 1%) and financial support (*n* = 1, 1%). Supporting internal factors included experiences of success (*n* = 2, 2%) as well as starting with the fun ones (*n* = 1, 1%).

Binary logistic regression revealed no significant effects of gender, age, or disease duration on participants’ responses (*p* > 0.05).

### Factors hindering lifestyle changes

Asking for factors that hinder PD patients from changing their lifestyle, many patients stated that they could only motivate themselves for a short period of time and then fell back into old habits (*n* = 39, 37%). Additionally, they also cited a lack of knowledge (*n* = 18, 17%) and lack of cooperation from their partners (*n* = 11, 10% partner does no sports; *n* = 13, 12% partner eats differently). In addition, some stated that they had physical limitations that hindered them from changing their lifestyle (*n* = 10, 9%). Lack of initial motivation (*n* = 5, 5%) as well as high food costs (*n* = 2, 2%) were rarely mentioned. Free-text responses (see Fig. 3) contained various hindering factors. Hindering external factors included lack of time because of job, family and care work (*n* = 8, 7%) as well as lack of knowledge and offerings (*n* = 4, 4%). Internal factors included motivational problems (*n* = 2, 2%), unhealthy food preferences (*n* = 2, 2%) as well as decrease of strength (*n* = 1, 1%).

In the analysis of potential clinical predictors using binary logistic regression models, gender emerged as a significant factor for the item assessing potential barriers to lifestyle change: female gender was associated with a higher likelihood of responding affirmatively to the statement that differing dietary habits of their partner would hinder them from changing their own lifestyle (*p* = 0.006). Moreover, older age was associated with endorsing lack of knowledge as a reason for not engaging in preventive behavior (Exp(B) = 1.077, 95% CI 1.012–1.147, *p* = 0.020), whereas longer disease duration was associated with a positive response to the statement to feel physically too limited to engage in exercise (Exp(B) = 1.120, 95% CI 1.023–1.226, *p* = 0.014). Beyond these findings, no significant associations were observed between gender, age, or disease duration and the participants’ responses.

## Discussion

The aim of this study was to gain insight into the relevance people with PD place on lifestyle changes and to investigate the factors that are considered helpful and hindering in implementing and maintaining lifestyle changes. The participants of this survey were highly interested in obtaining information about lifestyle changes that could potentially have a positive effect on the course of the disease. In addition, they suggested specific supportive measures that could help them implement lifestyle changes, such as prevention and bonus programs, digital solutions as well as support from their neurologist, family doctor and partners.

Currently, PD patients in Germany and many other countries do not receive organized support in implementing lifestyle changes. First comprehensive approaches on providing PD patients with structured care (visits to the local neurologists, regular examinations by a PD nurse as well as regular physiotherapy, occupational or speech therapy) in Germany have recently been tested and have already shown an improvement in the quality of life of PD patients [16]. However, these concepts did not include lifestyle measures. To take advantage of the possibility of influencing the course of the disease through lifestyle changes, it seems essential to develop structured programs that focus on this topic and can be offered to those affected.

For other lifestyle-related diseases such as diabetes, organized lifestyle interventions have been in place for years and have shown highly promising results in reducing blood sugar and improving insulin resistance as well as reducing diabetes-associated complications [17–20]. However, adherence to lifestyle changes is generally low or at best moderate if it is not accompanied by structured guidance and support [21, 22]. The benefits of guided lifestyle interventions have already been investigated in studies on other neurodegenerative diseases, particularly dementia. In this respect, the FINGER (Finnish Geriatric Intervention Study to Prevent Cognitive Impairment and Disability) and more recently the US POINTER (U.S. Study to Protect Brain Health Through Lifestyle Intervention to Reduce Risk) study have been excellent examples of how a guided multidomain lifestyle intervention that aims for high adherence can improve or at least maintain cognitive function in older individuals at risk of developing dementia in a time range of two years [23, 24]. Additionally, a recent randomized controlled trial confirmed the significant effects of a multidomain intervention on cognitive function in individuals with mild cognitive impairment or early Alzheimer dementia, with effects already evident after 20 weeks [25]. These studies underline the value of structured guidance to ensure a high level of adherence to lifestyle changes and thus enable a disease-modifying course.

The main challenges to utilizing lifestyle modifications as a disease-modifying treatment for PD are, firstly, the acceptance of lifestyle modifications as a form of treatment and, secondly, adherence to these lifestyle modifications. The results of this survey indicate that PD patients included here do accept lifestyle changes as a form of therapy for PD and consider them important. In addition, this survey revealed several important barriers that may prevent PD patients from implementing and maintaining lifestyle changes, which could be important for future patient programs in order to improve adherence to lifestyle measures. Patients stated that they needed more education and information about the importance and kind of lifestyle changes for PD. They would also like more guidance on how to implement these changes in everyday life. For example, the help of a dietician was mentioned as a supporting factor. This is in line with findings from previous studies which showed that a food literacy program (including meal planning and preparation) was able to improve dietary behavior of healthy participants [26, 27]. It should be noted that involving family members may be necessary to ensure effective implementation of healthy behaviors in daily life. In this study, 66% of respondents reported that partner involvement was important, whereas 12% indicated that differing eating habits of their partners hindered lifestyle changes. This response was more frequently observed among female participants, suggesting that future patient-oriented programs should consider potential gender differences and their impact on the adoption of healthy eating habits. The regression analyses further indicated that older patients were more likely to report being less informed about lifestyle measures, and patients with more advanced disease progression reported greater difficulties in implementing preventive strategies due to physical limitations. These findings highlight, on the one hand, that demographic factors such as age should not limit educational efforts, since prevention and support remain meaningful at every stage oft he disease; and, on the other hand, that offering preventive guidance early—before substantial physical limitations develop—is crucial to maximize patients’ ability to implement lifestyle recommendations. Taken together, patients included in this survey were already well educated about the possible effects of lifestyle changes. Still, structured guidance might optimize the effect in this already motivated patient group. In a previous study, patients indicated a desire for an integrative approach to PD management that included education about the disease as well as nutrition. Similar to our study, patients described a lack of structured information and little involvement of relatives. The authors therefore concluded that a comprehensive concept for the treatment of PD is urgently needed in Germany, which also contains sufficient information about the disease as well as its treatment [28]. In addition, patients in Germany have contact with both primary and secondary care providers, with patients in our survey considering specialized secondary care providers (neurologists in clinics or practices) to be particularly important for education and the implementation of preventive measures. It is therefore necessary to offer further training on this topic for both neurologists in training and specialists.

Finally, PD patients included in our study also stated that compatibility with other everyday tasks such as employment or family duties would be necessary for successful implementation. In particular, the repeated statement that the workload at their workplace is an obstacle to a change in lifestyle indicates a need for further research into occupational health management measures.

### Limitations

This study is based on a questionnaire, that was developed by experienced PD clinicians that has not yet undergone formal validation. Proceeding with this tool was chosen to enable accessible and timely patient participation, given the lack of evidence-based questionnaires addressing these specific aspects of PD. Validation and further refinement of the instrument remain essential next steps.

Patients were recruited exclusively from two specialized clinical settings. Due to the study design (distribution to all patients in the specified clinics during routine clinical practice), a systematic assessment of the response rate was not possible. Moreover, individuals attending a specialized outpatient or PD clinic are typically more motivated and better informed than the broader PD community and may be more willing to take responsibility for modifying lifestyle factors. Consequently, our study may overestimate patients’ motivation and knowledge in this area. Additionally, the questionnaire comprised only a limited number of items and did not include standardized clinical assessment for detailed characterization. As a result, we cannot provide data on educational background, socioeconomic status, living environment, access to healthcare services and potential comorbidities such as depression or anxiety. Cognitive and motor function were also not assessed, although these factors may influence both interest in and access to a healthy lifestyle. This may have led to an overestimation of compliance and engagement in our sample, highlighting the value of including these measures in future studies. Furthermore, the present analysis was conducted on a relatively small sample, and larger studies are needed to confirm these findings. Future research could also benefit from administering the questionnaire at multiple time points to assess whether patients’ responses, particularly regarding the information they have received, change over time.

## Conclusions

In summary, this study indicates that PD patients in this survey want to be informed about possible positive effects of changing lifestyle factors, and they already try to implement them into their lives. However, despite this high motivation, many patients reported factors that prevent them from changing their lifestyle, such as falling back into old habits due to a lack of motivation, lack of knowledge or habits of their partner not compatible with these lifestyle changes. The study shows initial clues that structured support for sustainable lifestyle changes, e.g. through prevention programs, digital solutions and partner involvement, might help PD patients achieve higher levels of adherence, which could be an important factor paving the way for more effective non-pharmacological disease modification. Due to its high relevance, brain health and its promotion through lifestyle changes has clearly become the focus of international initiatives in recent years, not only for primary or secondary prevention, but also for maintaining function and quality of life in already manifest neurological disorders [29, 30]. It is therefore surprising that in a disease such as PD, where there is clear evidence that lifestyle measures can positively influence the course of the disease, appropriate guidance has not yet played a major role in everyday clinical practice. This study shows once again that our patients are motivated to implement lifestyle changes and that the healthcare system should make more effective use of this important opportunity for disease modification.

## Supplementary Information

Additional file 1. Questionnaire on lifestyle changes. The Questionnaire on lifestyle changes used in this study translated into English.

## Data Availability

Data supporting the findings of this study are available on request from the corresponding author. The data is not publicly available due to privacy and ethical restrictions.

## Abbreviations

PD: Parkinson's disease
FINGER: Finnish Geriatric Intervention Study to Prevent Cognitive Impairment and Disability
US POINTER: U.S. Study to Protect Brain Health Through Lifestyle Intervention to Reduce Risk
