# Supplementary material for: Preventive measures for Parkinson’s disease: insights into motivation and barriers from the patients’ perspective
Source: BMC Neurol. 2026 Feb 11;26:134. doi: 10.1186/s12883-026-04703-0 (PMC12934006; doi:10.1186/s12883-026-04703-0)
Supplement: Supplementary file 1 — Additional file 1. Questionnaire on lifestyle changes. The Questionnaire on lifestyle changes used in this study translated into English. [file 12883_2026_4703_MOESM1_ESM.docx]

**General information**

**Diagnosis**

1. When were you diagnosed with Parkinson's disease?

________ (*please state the year*)

1. What medication are you currently taking?

| *Medication, e.g. Levodopa* | *Dosage, e.g. 3x a day 100mg* |
| --- | --- |
|  |  |
|  |  |
|  |  |
|  |  |
|  |  |
|  |  |
|  |  |
|  |  |
|  |  |
|  |  |
|  |  |

1. What other illnesses are you aware of?

________________________________________________________________________

________________________________________________________________________

________________________________________________________________________

________________________________________________________________________

________________________________________________________________________

________________________________________________________________________

________________________________________________________________________

**Advice on lifestyle aspects**

1. Have you been advised by your doctor that lifestyle changes (diet, exercise) can have a positive effect on the course of the disease?

 No  Yes

**If yes,** where were you told about this? *(multiple selection possible)*

 at the outpatient neurologist

 at the family doctor

 in a specialized outpatient clinic for Parkinson’s (e.g. university hospital)

 during a stay in a specialist Parkinson’s clinic

 in a rehabilitation clinic

 in another place: _____________

**If yes,** what were you told about? *(multiple selection possible)*

 Regular physical activity (sport) can have a positive influence on the course of the disease

 Aspects of nutrition (e.g. Mediterranean diet) can have a positive influence on the course of the disease

 Regular targeted training (physiotherapy, occupational therapy, speech therapy) can improve the symptoms of Parkinson's disease

 Other: ____________________________________________________

**If yes,** have you implemented the recommendations?

 No, I don't consider them important

 No, so far I haven't been able to bring myself to change anything

 No, from my point of view I already adhere to these lifestyle recommendations

 No, other reasons: ___________________________

 Yes (*multiple selection possible*):

 I pay more attention to sporting activity and physical exercise

 I pay more attention to my diet

 I am/was in physiotherapy, occupational therapy or speech therapy

1. How important do you consider that Parkinson's patients are informed about possible positive aspects of lifestyle changes when they are diagnosed?

**Very important**

**Not important aktiv**

**neutral**

1. How do you think Parkinson's patients should be informed about the relevance of lifestyle changes? (multiple selection possible)

 Public talks (e.g. in self-help groups)

 Oral information

 at the family doctor

 at the outpatient neurologist

 as part of a presentation at the University Hospital

 as part of a presentation at a Parkinson's specialist or rehabilitation clinic

 Written information

 at the family doctor

 at the outpatient neurologist

 as part of a presentation at the University Hospital

 as part of a presentation at a Parkinson's specialist or rehabilitation clinic

 Other: _______________________________________________

1. What could help you to make lasting lifestyle changes?

 Involvement of the partner

 Specific prevention programs, e.g. a sports program that I could participate in

 Bonus programs

 Digital solutions

 Individual advice and information from my family doctor

 Individual consultation and information from my neurologist

 Other: ___________________________________________________

1. What factors prevent you from changing your lifestyle? (*multiple selection possible*)

 I didn't know until now that this plays a major role in Parkinson's disease

 I cannot motivate myself

 I can only motivate myself for a short time and then fall back into old habits

 My partner does little exercise

 I feel too physically restricted to do sport

 My partner eats differently

 I get my food delivered and the choice is limited

 The cost of the recommended foods is too high

 Other: ___________________________________________________
